# Erratum

**DOI:** 10.1200/JCO.2016.72.0888

**Published:** 2017-02-01

The June 20, 2016, article by Choudhury et al, entitled “Afatinib Activity in Platinum-Refractory Metastatic Urothelial Carcinoma in Patients With ERBB Alterations” (J Clin Oncol 34:2165-2171, 2016), contained an error.

In the Acknowledgement section, University of Chicago’s Cancer Center Support Grant should have been listed. The Acknowledgement should have been given as: We thank the Human Tissue Resource **Center and the Cancer Center Support Grant** at the University of Chicago, and specifically Dr Shihong Li, for their support in performing the immunohistochemistry studies, and Heather Mashek for her assistance in scoring the fluorescence in situ hybridization assays.

The online version has been corrected in departure from the print. The authors apologize for the mistake.

